# Epidemiology of ambulance utilized patients in Addis Ababa, Ethiopia

**DOI:** 10.1186/s12913-018-3820-4

**Published:** 2018-12-27

**Authors:** Menbeu Sultan, Yonas Abebe, Assefu Welde Tsadik, Catherine Ann Jennings, Nee-Kofi Mould-Millman

**Affiliations:** 1 Department of Emergency Medicine and Critical Care, St. Paul’s Hospital Millennium Medical College, Addis Ababa, Ethiopia; 2 Department of Emergency Medicine and Critical Care Nursing, St. Paul’s Hospital Millennium Medical College, Addis Ababa, Ethiopia; 3 Federal Ministry of Health Ethiopia, Emergency and Critical Care Directorate, Addis Ababa, Ethiopia; 4 Columbia University Premedical Post baccalaureate, New York, USA; 5 University of Colorado School of Medicine, Aurora, USA

**Keywords:** Ambulance, Epidemiology, Inter-facility transfer

## Abstract

**Background:**

Well organized and appropriately utilized pre-hospital emergency services play a critical role in augmenting emergency care systems. The primary objective of this study was to understand the demographic and clinical profile of patients who used ambulances in Addis Ababa. The secondary objectives were to assess ambulance response time, transport time and reasons for referral amongst inter-facility transported patients in Addis Ababa.

**Methods:**

The study was designed as a cross-sectional retrospective chart review of ambulance transported patients using ambulance station records from Addis Ababa Fire and Emergency Prevention and Control Authority. With IRB approval, simple random sampling and manual review of six months of clinical records was performed. Data were collected by trained data collectors and descriptive analysis was done using SPSS version 20.

**Results:**

Female patients used ambulance services more often than males (female to male ratio of 3:1) and the mean age of the patients was 26 years. The most commonly transported age group was 16–30 years, followed by 31–50 years and neonatal patients (i.e. < 1 month). The majority of the patients had pregnancy related illnesses (*n* = 492, 61.4%), followed by general medical issues (*n* = 210, 26.2%) and injury secondary to trauma (*n* = 99, 12.3%). Most patients (*n* = 702, 87.6%) were transported for inter-facility transfers, while only 12.4% (n = 99) were primary responses (i.e. from the scene). Prolonged labor was the most common reason (*n* = 103, 23.4%) for inter-facility transfer of pregnant patients, followed by premature rupture of the amniotic membrane (*n* = 60, 13.6%). The mean dispatch to scene time interval was 10.1 min, and mean scene to facility time interval was 17.2 min.

**Conclusion:**

Inter-facility transfers accounted for the largest proportion of ambulance utilization and dispatch in Addis Ababa. Ambulance transport time was twice as long compared to international recommendations of less than eight minutes for emergent transports. The most common reasons for ambulance dispatch were Obstetric. We recommend urgent action to decrease the transport times and to dedicate further pre-hospital resources to address the high burden of inter-facility transfers.

## Background

Emergency Medical Service (EMS) systems exist to reduce death and disability for those experiencing life or limb threatening medical emergencies in the pre-hospital environment. A primary operational task of EMS systems is to provide out of hospital medical care and transport to the most appropriate health facility for further care. A meta-analysis by Jaymie *et. al* in developing nations has shown a 25% decreased risk of death from trauma in areas that had a pre-hospital trauma system [1]. Furthermore, if appropriately used, ambulances are highly cost effective in reducing mortality from emergency conditions [2, 3].

Given the epidemiological shift from communicable diseases to non-communicable diseases occurring in Ethiopia, there is an increasing demand for EMS systems [4]. In one community-based study done from 2006 to 2009 in Addis Ababa, cardiovascular diseases accounted for 24% of deaths. Although there has been a significant reduction in pregnancy related mortality, non-communicable diseases like heart disease, stroke, and respiratory disease are increasing [4, 5]. In postmortem examination of sudden deaths in Addis Ababa, 52% of the individuals died from natural causes and 48% died from injury-related events. In the injury related group, road traffic injury (RTI) was the most common cause of accidental death (80%), and no pre-hospital care was received [4, 6]. Road traffic injury is steadily increasing and is disproportionately affecting the economically active groups (i.e. those 15–59 years of age) [7–9].

Response time, availability and training of pre-hospital providers, available resources, and the appropriateness of calls contribute to the overall quality of pre-hospital care. A common benchmark response time in high-income settings has been recommended to be less than eight minutes [10], but no evidence based equivalent exists for low and middle income settings. Addis Ababa ambulance service has a fragmented organizational structure with very limited number of ambulance vehicles [11]. Similar to reports from other African countries, in Addis Ababa the use of private cars and taxis is the current norm for transporting an emergency patient from scene to health facility [12].

In past 10 years, the Federal Ministry of Health (FMoH) has introduced an effort to improve the EMS systems in Ethiopia. Efforts include distribution of ambulances to all regions, providing at least one ambulance per district (woreda), training of paramedics, and procurement of on-board medical equipment [13]. Addis Ababa Fire and Emergency Prevention and Control Authority (AAFEPCA), along with few private companies, provide the major pre-hospital emergency services care for the city. Under their authority, there is one central dispatch center for fire and pre-hospital services, eight ambulance stations, and about 32 ambulances.The authority provides free pre-hospital care, including scene to health facility and inter-facility transfers. A free ambulance phone number (939) is used for access by the public. The care providers are nurses with short term pre-hospital patient care training. Although the pre-hospital service has existed for over 10-years, the epidemiology of patients using ambulances and the appropriateness of this usage remains unstudied. This study aims to examine patients’ clinical characteristics and reasons for ambulance use in Addis Ababa.

## Objective

The primary objective of this study was to assess the demographics and clinical profiles of patients utilizing ambulances in Addis Ababa. The secondary objectives of the study were to assess ambulance response time, transport time, and reasons for referral amongst inter-facility transported patients in Addis Ababa.

## Methods

### Study setting and design

This study was conducted in Addis Ababa, the capital city of Ethiopia. The city has a population of more than four million people across ten sub-cities and is a site for diplomats and international organizations including the African Union and the Economic Commission for Africa. Nearly 70% of the population is younger than 30 years of age, and there is a slightly higher number of females (F:M 1.03:1). Addis Ababa Fire and Emergency Prevention and Control Authority is a government funded organization delivering free emergency and ambulance services for the public. At the time of the study, it had eight ambulance stations distributed indifferent sub-cities of the town. A cross-sectional retrospective chart review of patient records was conducted from the AAFEPCA ambulance stations.

### Sample size and sampling

Four ambulance stations were randomly chosen using computer generated numbers from the available eight stations. The selected ambulance stations were Addis Ketema, Bole, Kirkos and Nifas Silk-Lafto. Each ambulance station maintained written records of ambulance transported patients. Based on data from a similar study done in Uganda, it was anticipated that 66% of ambulance utilization was for pregnancy related conditions [14], and sample size was calculated using the single proportion *P* = 0.66 (where P is the proportion of patients with pregnancy related conditions), with 95% confidence interval, 4% of degree of freedom, and 5% added for exclusion of incomplete data. The calculated sample size of 565 was multiplied by 1.5 for the cluster effect to obtain the final sample size estimate of 847. Patient charts were selected from paper based patient registry (at the individual ambulance stations) using simple random sampling.

### Data collection and analysis

The data abstraction format was prepared and pre-tested. Once patient charts were identified, study investigators screened the charts for completeness. Data were collected by data collectors trained by study investigators and the data quality was controlled by the investigators throughout the data collection time by doing double chart reviews. Data cleaning and analysis for descriptive statistics was done using SPSS version 20. Missing data were interpreted as it was without replacement.

### Ethical considerations

The study protocol was reviewed and approved by St. Paul Hospital Millennium Medical College Institutional Review Board (IRB). Confidentiality was maintained throughout the study.

## Results

In a six-month period from July to December of 2016, a total of 7800 patients’ clinical reports existed from the four ambulance stations tested. Of those, 847 patient records were sampled and 46 records were then excluded due to incomplete data. Of the final 801 study participants, (*n* = 605, 75.5%) were female, and patients aged 16–30 years were the most common age group represented (*n* = 583, 72.7%). The most common location of patients for initiation of ambulance transport was a health facility (*n* = 702, 87.6%) and public hospitals were the most common destination facility (*n* = 726, 90.6%). The mean ambulance response time interval was 10.1 min and the mean patient transport time interval was 17.2 min (Table 1).

**Table 1:** Characteristics of ambulance used patients in Addis Ababa stratified by type of illness

Patient characteristic	Number of patients with injury	Percentage	Number of patients with disease	Percentage	Number of patients with pregnancy related condition	Percentage	Total
Age of the patients in years							
0–1	0	0	32	100	NA	NA	32
2–5	2	12.5	14	87.5	NA	NA	16
6–15	5	31.3	11	68.8	NA	NA	16
16–30	62	10.6	88	15.1	433	74.3	583
31–50	22	18.6	37	31.4	59	50.0	118
> 50	8	22.2	28	77.8	0	.0	36
Sex							
Male	77	39.3	119	60.7	NA	NA	196
Female	22	3.6	91	15.0	492	81.3	605
Scene location							
Health facility	80	11.4	176	25.1	446	63.5	702
Home	3	5.3	11	19.3	43	75.4	57
Industry	1	11.1	7	77.8	1	11.1	9
Road	13	46.4	14	50.0	1	3.6	28
Work place	0	0	2	66.7	1	33.3	3
Other	2	100	0	.0	0	0.0	2
Minutesfrom dispatch to scene location							
0–9	57	11.7	139	28.5	291	59.8	487
10–19	23	10.3	48	21.6	151	68.0	222
20–39	16	18.8	23	27.1	46	54.1	85
> −40	3	42.8	0	0.0	4	57.1	7
Minutes from scene to destination							
0–9	13	7.5	51	29.3	110	63.2	174
10–19	40	12.8	61	19.6	211	67.63	312
20–39	39	15.5	73	28.9	140	55.6	252
> 40	6	22.2	13	48.2	8	29.6	27
Receiving health institute							
Public hospital	84	11.6	184	25.3	458	63.1	726
Public health center	15	20.0	26	34.7	34	45.3	75

*NA* not applicable

### Clinical characteristics of the patients

The majority of patients had pregnancy related illnesses (*n* = 492, 61.4%), followed by medical illness such as respiratory distress (*n* = 210, 26.2%) and trauma (*n* = 99, 12%). On primary assessment of the patients, eight (1%) patients had airway problems, 16(2%) patients had breathing problems, and 77(9.8%) of patients had severe bleeding. On the assessment of mental status, the majority of the patients were fully alert, (*n* = 651, 81.6%). Regarding vital signs, 82(13.1%) patients had hypotension and 125(18.6%) patients had tachypnea. Intravenous fluid administration was the most common on-board treatment given (*n* = 311, 39%) (Table 2).

**Table 2:** Clinical data of ambulance transported patients in Addis Ababa, Ethiopia

	Parameter	Number of patients with condition	Percentage
Air way (*n* = 798)	Clear	790	99.0
	Partly blocked	8	1.00
Breathing (*n* = 798)	Effective	782	98.0
	Ineffective	16	2.00
Severe bleeding (*n* = 798)	Yes	77	9.60
	No	721	90.4
Respiratory rate for age^a^ (*n* = 673)	Tachypnea	125	18.7
	Normal	537	79.8
	Bradypnea	11	1.6
Pulse rate for age^a^ (*n* = 711)	Fast	46	6.5
	Normal	651	91.6
	Slow	14	1.90
Blood pressure^a^ (*n* = 616)	Normal	534	86.7
	Low	82	13.3
Casualty responsiveness(*n* = 798)	Alert	651	81.6
	Verbal response	112	14.0
	Responds to pain	22	2.76
	Unresponsive	13	1.6
Treatment given in the ambulance (*n* = 485)	IV line	311	64.1
	Oxygen	107	22.1
	Bleeding Arrest	62	12.8
	Deliver care	3	0.6
	CPR	2	0.4

Key:^a^Low blood pressure for adults < 90/60mmgh, Normal systolic 90-120mmgh.fast pulse rates for adults > 110 per minute, Brady cardia is heart rate less than 60 per minute, tachypnea is RR > 22 per minute for adults

### Inter facility transfer of patients

**Fig. 1:**
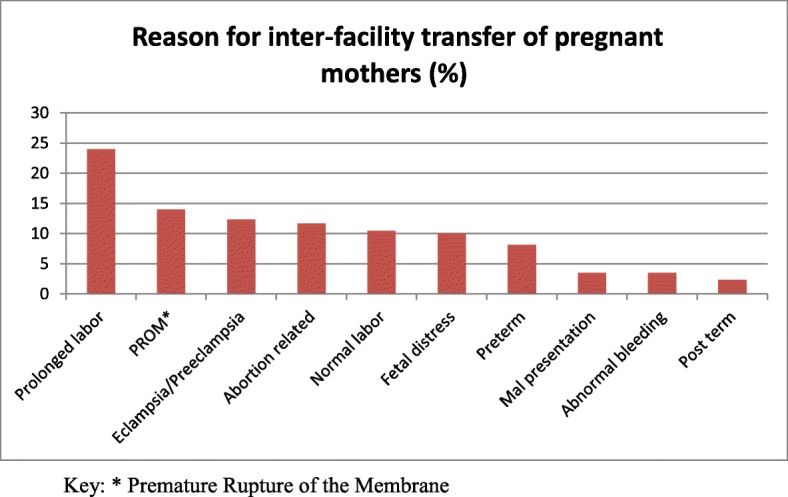
Common pregnancy related illnesses requiring inter facility ambulance transportation in Addis Ababa

**Fig. 2:**
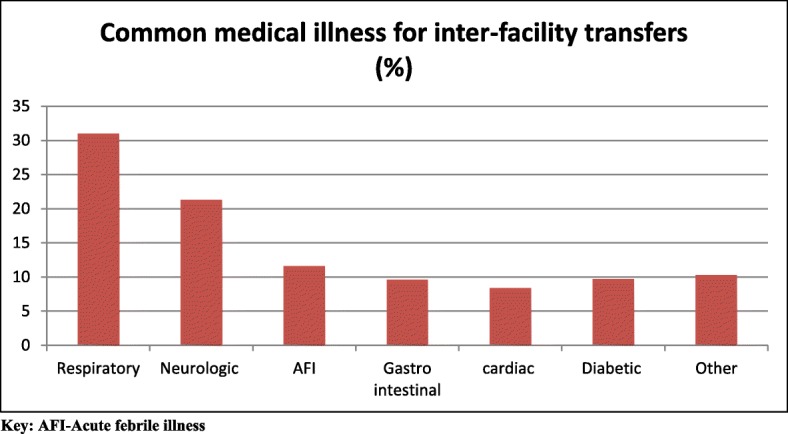
Common medical illness requiring ambulance inter-facility transfer in Addis Ababa

Of all study participants ambulance transportation, the majority were inter-facility transfers (*n* = 702, 87.6%). Health centers (the lowest level of public health facility in Addis Ababa) were the most common referring health institutions, accounting for 82.6% of cases (*n* = 578), followed by public hospitals (*n* = 103, 14.7%). Pregnancy related causes were the most common reasons for inter facility transfer (*N* = 446, 63.5%). Prolonged labor (*n* = 103, 24.0%) was the most commonly stated reason for pregnancy related inter-facility transfer, followed by premature rupture of amniotic membrane (PROM) (*n* = 60, 14.0%) (Fig. 1). Non-pregnancy related causes (i.e. trauma and medical) of inter-facility transfer account for 36.5% of cases (*n* = 256). Of these non-obstetric inter-facility transfer cases, trauma accounted for 30% (*n* = 80), general surgical patients for 7.8% (*n* = 20), sick newborns for 8.8% (*n* = 23), and the remaining 52.0% (*n* = 133) had medical illness. Figure 2 details common medical illness for inter-facility transfer.

## Discussion

During the surveyed time period, pregnancy related emergencies were the most common reasons for ambulance use in Addis Ababa (61.4%). Additionally, the majority of ambulance use was to conduct inter-facility transfer of patients. On primary survey, severe bleeding was found as the most common problem accounting for 77 cases (9.6%), followed by breathing problems. The most common non-obstetric referral was injury, which accounted for about one-third of all transports. A prior study in Addis Ababa identified that 41.2% of the injuries occurred on the road and 12.0% at home [6]. The majority (81.4%) of the patients did not receive post-crash care at the pre-hospital level and 41.2% died outside the hospitals [6, 15]. Moreover, our finding showed ambulances are mainly being used for inter-facility transfer of patients; these findings show indicate there should be an action to shift the service focus of ambulances to conduct more primary (scene) responses.

In a similar report from the Ashanti Region in Ghana, 76% of all national cases were inter-facility transports [12]. A study by Stewart de Ramirez et al. in Uganda showed that 66% of inter-facility transfers were obstetric related and 77.8% were for female patients [14]. Furthermore, 52% of all obstetric transfers and 62% of all trauma transfers were from low level to high level hospital care [14]. These African studies, including this study in Addis Ababa, show a high burden of ambulance use for inter-facility transfers [16]. In addition, there are reports of secondary over-triage, including the unnecessary referral of patients with mild conditions to a higher level of care facility, leading to potentially un-necessary transfer of patients. A recent study done in the Kwazulu-Natal district of South Africa reported an in appropriate dispatch of resource, where more than 58% of cases required either no intervention or transport only [17, 18]. Our study results may indicate the necessity for increasing the capacity of the health centers and providers to prevent unnecessary inter-facility transfers in the study settings.

Amongst pregnancy related transfers, prolonged labor was the most commonly stated reason for transfer (*n* = 103, 24.0%), followed by premature rupture of amniotic membrane (*n* = 60, 14.0%). Additionally, postpartum hemorrhage accounted for 15 cases (4%) of obstetric transfers and neonatal emergencies accounted for 23 cases (8.8%) of non-obstetrictransfers. In a 2013 study of Addis Ababa health centers, poor provider competency was reported, with insufficient knowledge for diagnosing postpartum hemorrhage (PPH) and birth asphyxia, as well as poor skills in neonatal resuscitation [19]. Given that the majority of transfers were from health centers to hospitals, there may be a need for capacity building in the health centers to decrease the need for referrals. In addition, unnecessary transfer of patients, like those who have PROM, can be reduced by implementing an obstetric ambulance referral protocol and improving training of health care providers in health centers.

Most ambulance used patients are pregnant mothers having prolonged labor when time is crucial to decrease both maternal and fetal morbidity and mortality. The response time for ambulances is an important quality of ambulance care for time sensitive emergency conditions. Results identified that the ambulances in Addis Ababa are spending more than nine minutes to reach the scene. However, research shows the mortality risk for patients with ambulance response time of more than five minutes is three times higher than those less than five minutes [20]. The estimated effect of a one minute reduction in response time shows improved odds of survival by 24% [21].

One method for decreasing response time is increasing the number of available ambulances, although in one South African study, only increasing the number of ambulances did not help meet the urban priority response time goals [22]. In another South African study, emergency ambulance location had a greater effect on the response time performance [23]. Yet another study showed that increasing the ambulance base and using satellite deployment with variations according to demand and time of the day significantly decreased the median monthly response time and the proportion of patients transported in less than eight minutes [24].

There were eight ambulance stations in Addis Ababa at the time of this study. In addition to improving the road for ambulances, the base and distribution of the ambulances should also be evaluated and improved to decrease the response time. For example, dynamic postings, increasing the number of ambulances, and allocating ambulances to prioritize inter-facility transfers versus primary/scene responses can improve the disproportionate utilization of ambulance for inter-facility transfer.

### Limitations of the study

The findings and interpretations are limited by the retrospective nature of the chart review and instances of missing data. The study involved only the capital city which makes findings and recommendations difficult to generalize to other non-urban metropolitan areas in Ethiopia.

## Conclusion

Inter-facility transfer of patients account for the predominant proportion of ambulance dispatches in Addis Ababa. Ambulances had relatively prolonged response and transport time intervals compared to international high-priority benchmarks. The common causes for ambulance dispatch were pregnancy related emergencies. Action is needed to decrease the transport times and to dedicate further resources to address the high burden of inter-facility transfers.

## Abbreviations

AAFEPCA: Addis Ababa fire emergency prevention and control authority
*ED*: Emergency department
EMS: Emergency medical services
*RTI*: Road traffic injury
*SPSS*: Statistical package for the social sciences
